# MeCP2 Affects Skeletal Muscle Growth and Morphology through Non Cell-Autonomous Mechanisms

**DOI:** 10.1371/journal.pone.0130183

**Published:** 2015-06-22

**Authors:** Valentina Conti, Anna Gandaglia, Francesco Galli, Mario Tirone, Elisa Bellini, Lara Campana, Charlotte Kilstrup-Nielsen, Patrizia Rovere-Querini, Silvia Brunelli, Nicoletta Landsberger

**Affiliations:** 1 Division of Regenerative Medicine, Stem Cells and Gene Therapy, San Raffaele Scientific Institute, Milan, Italy; 2 San Raffaele Rett Research Unit, Division of Neuroscience, San Raffaele Scientific Insitute, Milan, Italy; 3 University of Insubria, PhD School in Neurobiology, Busto Arsizio, Italy; 4 Department of Health Sciences, University of Milano-Bicocca, Monza, Italy; 5 Division of Immunology, Transplantation and Infectious Diseases, San Raffaele Scientific Institute, Milan, Italy; 6 Vita-Salute San Raffaele University, School of Medicine, Milan, Italy; 7 Laboratory of Genetic and Epigenetic Control of Gene Expression, Division of Biomedical Research, Department of Theoretical and Applied Sciences, University of Insubria, Busto Arsizio, Italy; 8 Department of Medical Biotechnology and Translational Medicine, University of Milan, Segrate Italy; Institute of Genetics and Biophysics, ITALY

## Abstract

Rett syndrome (RTT) is an autism spectrum disorder mainly caused by mutations in the X-linked *MECP2* gene and affecting roughly 1 out of 10.000 born girls. Symptoms range in severity and include stereotypical movement, lack of spoken language, seizures, ataxia and severe intellectual disability. Notably, muscle tone is generally abnormal in RTT girls and women and the *Mecp2*-null mouse model constitutively reflects this disease feature. We hypothesized that MeCP2 in muscle might physiologically contribute to its development and/or homeostasis, and conversely its defects in RTT might alter the tissue integrity or function. We show here that a disorganized architecture, with hypotrophic fibres and tissue fibrosis, characterizes skeletal muscles retrieved from *Mecp2*-null mice. Alterations of the IGF-1/Akt/mTOR pathway accompany the muscle phenotype. A conditional mouse model selectively depleted of *Mecp2* in skeletal muscles is characterized by healthy muscles that are morphologically and molecularly indistinguishable from those of wild-type mice raising the possibility that hypotonia in RTT is mainly, if not exclusively, mediated by non-cell autonomous effects. Our results suggest that defects in paracrine/endocrine signaling and, in particular, in the GH/IGF axis appear as the major cause of the observed muscular defects. Remarkably, this is the first study describing the selective deletion of *Mecp2* outside the brain. Similar future studies will permit to unambiguously define the direct impact of MeCP2 on tissue dysfunctions.

## Introduction

Rett syndrome (RTT; MIM 312750) is a genetic disorder that because of its prevalence (roughly 1/10.000 born girls) is considered one of the most frequent causes of intellectual disability in females worldwide [1]. Typical RTT patients appear to develop normally throughout the first 6–18 months of life, when neurological development arrests and a regression phase occurs, leading to the loss of previously acquired skills. During and after the regression phase, patients develop typical symptoms including continuous stereotypic hand movements with a decline of purposeful hand use, loss of language skills, the appearance of autistic features, gait abnormalities, breathing irregularities, seizures, scoliosis and autonomic dysfunctions [2]. Mild generalized hypotonia is frequently observed in the first months of life of RTT patients, when symptoms are not yet overt. Moreover, an abnormal muscle tone is generally observed at later times [3]. Accordingly, abnormal muscle tone is a supportive criterion for the clinical diagnosis of atypical RTT [3].

Most girls affected by RTT carry *de novo* mutations in the X-linked *MECP2* gene [4]. The causative role of *MECP2* in RTT has been further supported by mouse models carrying *Mecp2* alterations [5]. *Mecp2*-null males (*Mecp2* ^*-/y*^) have no apparent phenotype up to 3–8 weeks of age, when they develop gross abnormalities, such as locomotor defects, hindlimb clasping, tremors, breathing abnormalities, seizures, reduced spontaneous movements and severe hypotonia. Symptoms worsen over time and the animals die within 6–10 weeks of age [5].

Since *MECP2* codes for an epigenetic transcriptional regulator that, although ubiquitously expressed, is particular abundant in brain [6] and since RTT has always been considered a pediatric neurological condition, conditional knock-out mice have been generated in order to understand the role of *Mecp2* in discrete brain regions or cell types. Overall, these studies have supported the contention that RTT is mainly, if not exclusively, a neuronal disease. However, the demonstration of increased glial gene expression in post-mortem female RTT brains and an altered glial metabolism in *Mecp2* mouse models [7] have prompted the study of a possible role of glia in MeCP2-related conditions. It has been demonstrated that *Mecp2*-null astrocytes affect neuronal growth and health in vitro and mouse breathing and locomotor activity in vivo [7]. Further, a role for microglia in RTT has been suggested by the benefit of transplanting wild-type bone marrow stem cells into irradiated young *Mecp2-*null mice [8]. Although no conditional mouse models inactivating *Mecp2* in organs different from brain have yet been generated, a role for MeCP2 in the development of heart and skeleton has been proposed [9].

In light of these considerations and of the severe hypotonia affecting RTT patients and mice, we investigated whether MeCP2 expression is required for the development and homeostasis of the skeletal muscle using two mouse models. The results demonstrate that the muscle of the *Mecp2*-null mouse suffers of a severe hypotonia that is not directly mediated by the lack of MeCP2 in this tissue.

## Results

### MeCP2 deficiency determines severe skeletal muscle atrophy and the deregulation of the IGF/AKT signaling pathway

**Fig 1:**
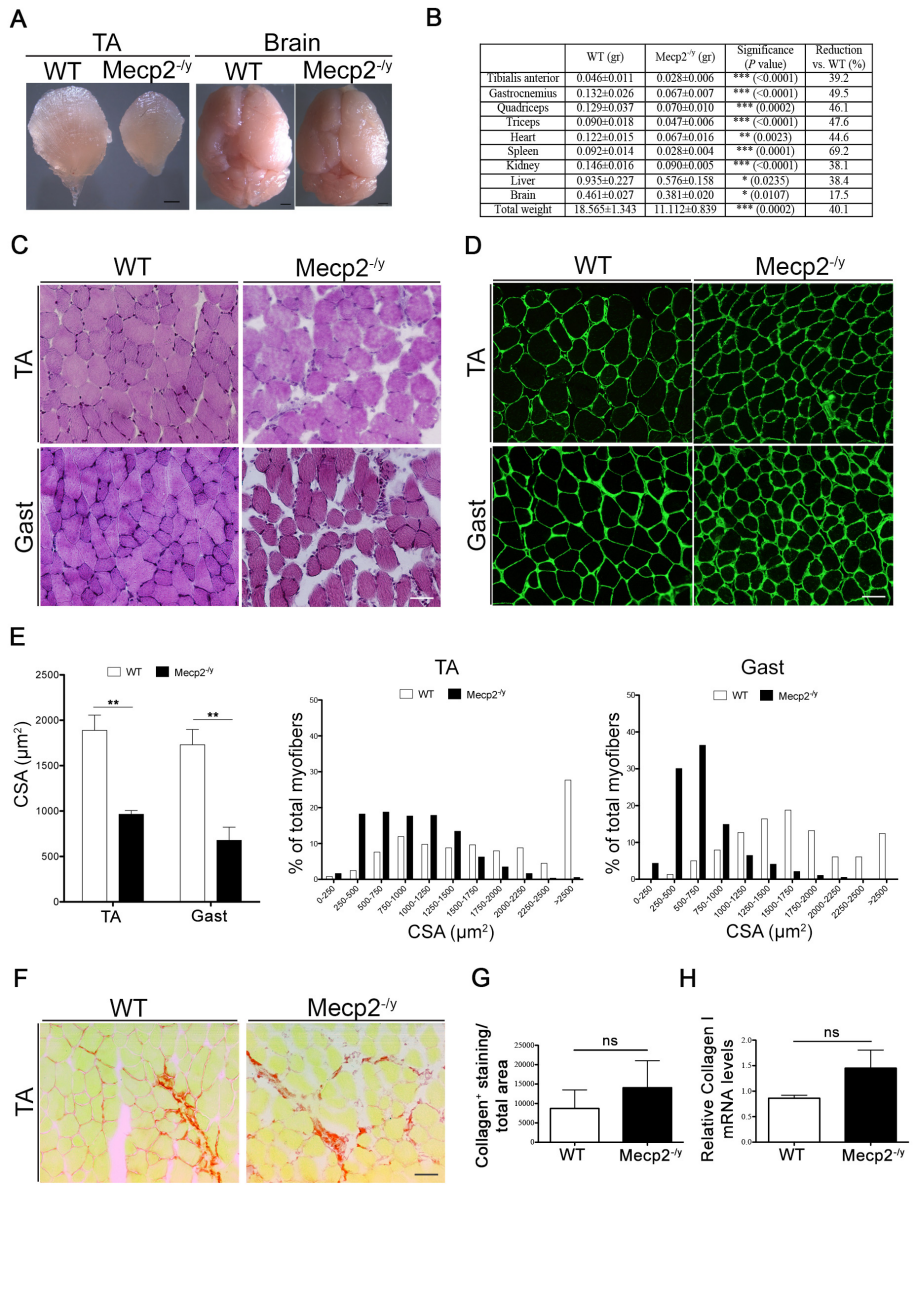
*Mecp2* ^*-/y*^ mice exhibit skeletal muscle disorganization and atrophy. (A) Representative pictures of TA and brain of 6 weeks old WT and *Mecp2* ^***-/y***^ mice. Scale bars = 0,5 mm. (B) Measures of organ weights are indicated as means ± s.e.m. Unpaired *t*-test was performed and *P* values are shown. Table also reports the percentage of reduction observed in *Mecp2* ^***-/y***^ mice (n≥6), calculated considering WT values as 100%. (C) Representative TA and gastrocnemius (Gast) muscle sections stained with H&E. Scale bar = 50 μm. (D) Representative TA and gastrocnemius muscle sections immunostained for Laminin. Scale bar = 50 μm. (E) CSA analysis of single muscle fibers, showing the abundance of smaller fibers in *Mecp2* ^***-/y***^ mice compared to WT mice. At least 150 myofibers were counted for each animal/muscle, n = 3 per genotype. Left panel: quantification of mean CSA ± s.e.m. Significance is calculated with *t* test (**, *P* value: 0.0059 for TA; **, *P* value: 0.0089 for Gast). Central and right panels: size distribution of single muscle fibers. *x*-Axis = fiber size (μm^***2***^); *y*-Axis = percentage of fibers. (F) TA sections (n = 3) were stained with Sirius Red and the total amount of collagen was quantified with ImageJ (G); ns, *P* value: 0.5593. (H) *Collagen I* mRNA expression was assessed in WT and *Mecp2* ^***-/y***^ gastrocnemius (n = 3). Significance is calculated with *t* test (ns, *P* value: 0.1126).

*Mecp2*-null mice show evident muscle hypotonia, postural defects and locomotor deficits. To investigate possible skeletal muscle defects associated with these features, we initially verified the integrity and the structure of the tissue. We retrieved muscles and other organs from 6 weeks old *Mecp2* ^*-/y*^ mice [10] and wild-type (WT) littermates. *Mecp2-*null muscles analyzed were much smaller than those of WT littermates (39.2% to 49.5% weight reduction ± s.e.m., Fig 1A and 1B). The reduction is in line with the overall reduced body weight of this *Mecp2* mouse model (Fig 1A and 1B).

To investigate the overall organization of the *Mecp2*-null muscle, we analyzed the histological and histochemical features of the tissue after staining of muscle sections with Hematoxilin & Eosin. As shown in Fig 1C, *Mecp2*-null skeletal muscles are characterized by a disrupted architectural structure (Fig 1C). No necrotic or regenerating centronucleated fibers were observed, indicating that RTT muscles are not dystrophic. Muscle fibers lacking MeCP2 have a reduced cross section area (CSA), which is consistent with reduced muscle mass (Fig 1D and 1E). Sirius Red staining (Fig 1F and 1G) and collagen I mRNA expression (Fig 1H) indicate a tendency of increased accumulation of fibrotic tissue in *Mecp2-*null muscles, even if not statistically significant.

Most skeletal muscles contain a mixture of different types of myofibers; in particular, type I myofibers are slow twitch, and fatigue resistant, whereas type II are fast twitch, either moderately fatigue-resistant (IIA) or not fatigue-resistant (IIB). To address whether *Mecp2* deficiency might affect fiber-type composition we tested immunoreactivity to myosin heavy chain I (MHCI) and IIb (MHCIIB), respectively used as markers of slow and fast fibers. Analyses were performed in adult gastrocnemii; as shown in S1 Fig, no difference was revealed.

Muscle atrophy in the absence of overt necrosis could be caused by abnormal innervation and/or functioning of the neuromuscular plaque. The number, shape and density of the neuromuscular junctions are comparable between WT and *Mecp2* ^*-/y*^ mice at 5 weeks of age, as assessed by immunofluorescence (S2 Fig), suggesting that the overall morphology of the neuromuscular plaque is maintained in the absence of MeCP2. This is in accordance with earlier studies demonstrating that in RTT subjects motor action potentials could be evoked easily following electromagnetic stimulation of the motor cortex [11], therefore confirming that innervation is functional.

**Fig 2:**
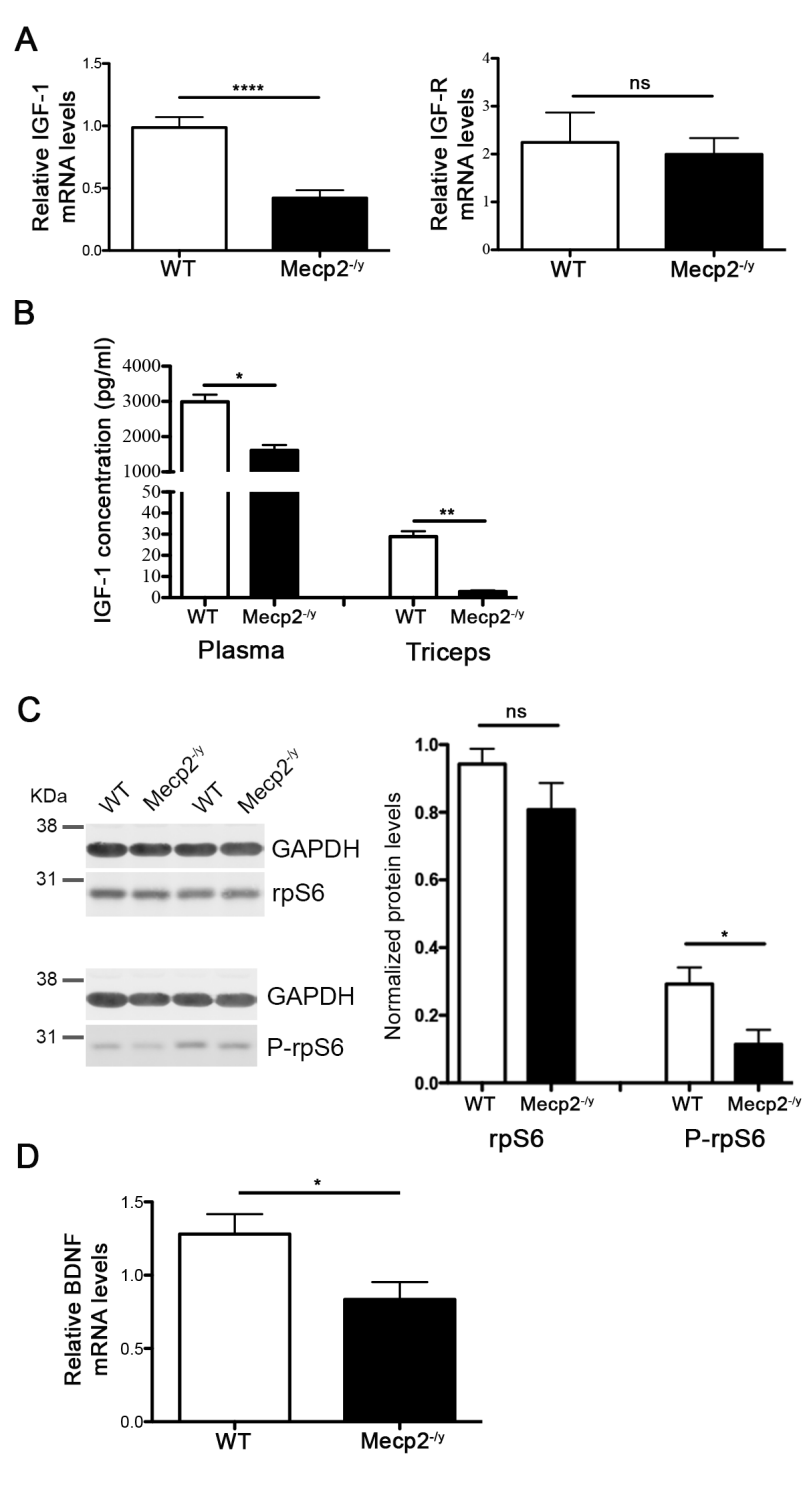
*Mecp2* ^*-/y*^ muscles exhibit deregulated protein synthesis signalling pathways. (A) qPCR evaluation of *IGF-1* (n = 8) and *IGF-R* (n = 3) mRNA expression in WT and *Mecp2* ^***-/y***^ gastrocnemius. All data points were calculated in triplicate, normalized to *GAPDH* and represented as gene expression relative to WT expression. Significance is calculated with *t* test (**** *P* value: <0.0001 for *IGF-1*; ns, *P* value: 0.7437 for *IGF-R*). Data are represented as mean ± s.e.m. (B) ELISA assay quantifying IGF-1 protein levels on WT and *Mecp2* ^***-/y***^ plasma and triceps (n = 6). All data points were calculated in duplicate. Significance is calculated with *t* test (*, *P* value: 0.0313 and **, *P* value: 0.0096). Data are represented as mean ± s.e.m. (C) Representative WB (left) and summary graph (right) of total rpS6 and phosphorylated rpS6 protein levels in WT and *Mecp2* ^***-/y***^ triceps lysates (n = 6). GAPDH was included as loading control. Significance is calculated with *t* test (*, *P* value: 0.0225; ns, *P* value: 0.1924). Data are represented as mean ± s.e.m. (D) qPCR evaluation of *BDNF* mRNA expression in WT and *Mecp2* ^***-/y***^ Gast. All data points were calculated in triplicate, normalized to *GAPDH* and represented relative to the WT expression (n = 5). Significance is calculated with *t* test (*, *P* value: 0.0425).

The deregulation of signaling pathways involved in myofiber maintenance and homeostasis and in the balance between protein synthesis and degradation could underlie muscle hypotrophy. Insulin-like Growth Factor 1 (IGF1) in particular has been suggested as a therapeutic agent for RTT [12–15] and clinical trials are ongoing in patients; further, MeCP2 affects protein synthesis and the AKT/mTOR pathway in brain [16]. Accordingly, we demonstrate that IGF1 mRNA expression is down regulated in *Mecp2*-null muscles retrieved from 6 weeks old mice (Fig 2A, *P*<0.0001) while the levels of the IGF1-receptor are unaffected (Fig 2A). IGF-1 protein expression is significantly reduced in *Mecp2* ^*-/y*^ mice, both in the blood and in the muscle (Fig 2B, *P* = 0.0313, *P* = 0.0096, respectively). The expression of phosphorylated ribosomal protein S6 (P-rpS6), involved in protein synthesis and down-regulated in brains of MeCP2 defective mice and in human neurons derived from iPS cells [16,17], is also lower in MeCP2 deficient muscles with respect to wild-type controls (Fig 2C).

Although neurotrophins are best known for their functions in regulating neuronal survival, plasticity and growth, it is now evident that they play important roles in several tissues. Among these, skeletal muscle is an abundant source of neurotrophic support during development and expresses several neurotrophin receptors, providing the basis for neurotrophin signaling in skeletal muscle [18]. In particular, BDNF is expressed in skeletal muscle under various physiological and pathological conditions [19], where it affects proliferation and differentiation of primary mioblasts and myotube size [20]. A functional interaction between MeCP2 and BDNF in brain has been demonstrated, leading to a reduced expression of BDNF in the brain of *Mecp2* mutant mice [21]. Thus, we addressed whether the neurotrophin expression might be also altered in *Mecp2-*null muscles. Here we show that the expression of *Bdnf* is significantly reduced in muscles of 6 weeks old *Mecp2*-null mice (Fig 2D)

### MeCP2 deficiency affects muscle fiber growth during regeneration

**Fig 3:**
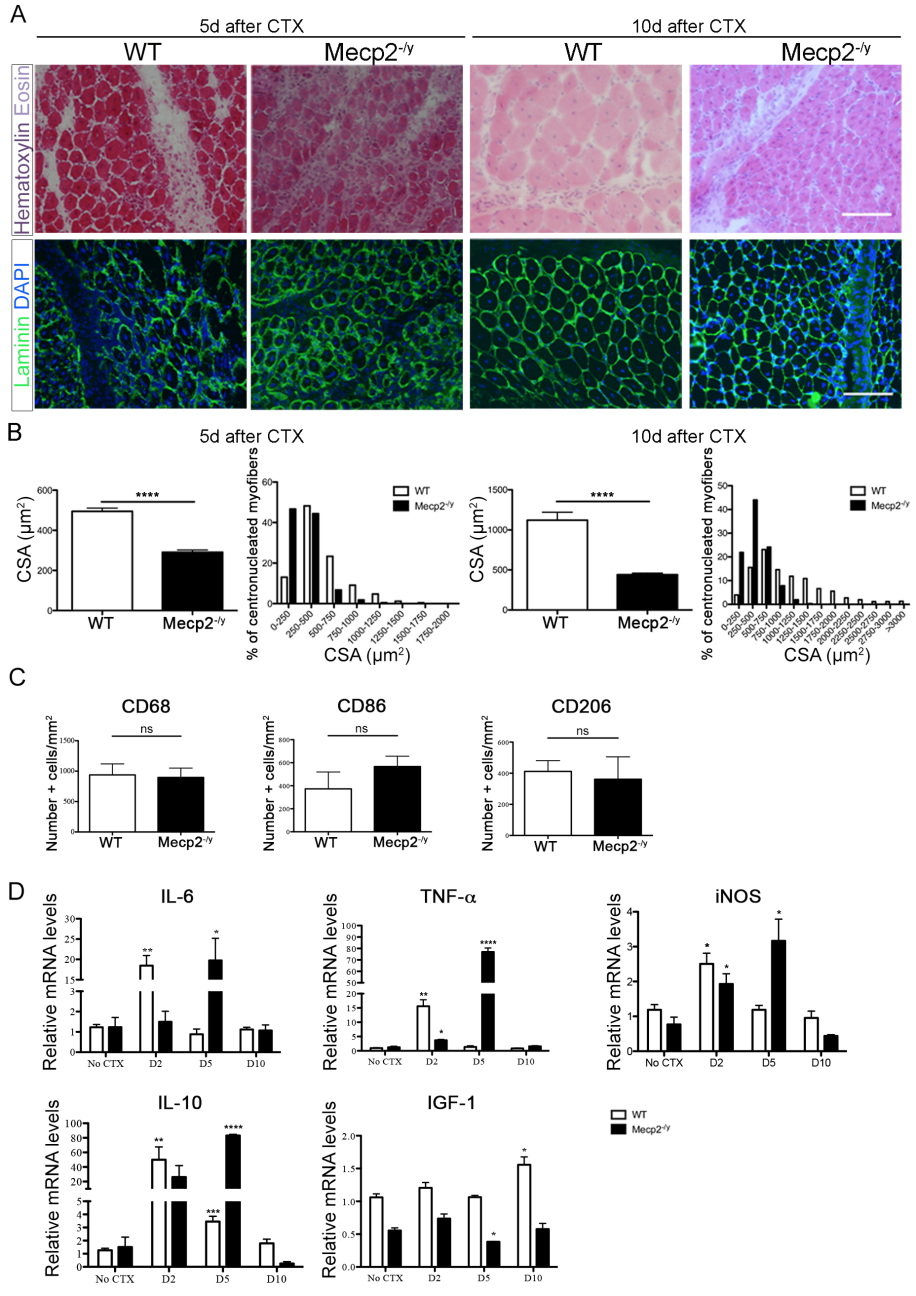
Regeneration after acute muscle damage in *Mecp2* ^*-/y*^ skeletal muscle. (A) Representative gastrocnemius cross-sections of WT and *Mecp2* ^***-/y***^ mice 5 days (left panels) and 10 days (right panels) after Ctx injury stained with Hematoxylin and Eosin or immunostained for Laminin/DAPI to recognize centrally-nucleated myofibers. Scale bar = 100 μm. (B) Mean CSA ± s.e.m. and distribution of regenerating centronucleated myofibers 5 days (left panel) and 10 days (right panel) after Ctx injury. At least 150 myofibers were counted for each animal; significance is calculated with *t* test (****: p<0.0001). (C) Quantification of the number of CD68^***+***^, CD86^***+***^ and CD206^***+***^ cells located in the damaged area of WT and *Mecp2* ^***-/y***^ gastrocnemii 5 days after Ctx injury. Significance is calculated with *t* test (ns, CD68^***+***^: *P* value 0.8650; CD86^***+***^: *P* value 0.3513; CD206^***+***^: *P* value 0.7421. n≥3). Data are represented as mean ± s.e.m. (D) qPCR evaluation of *IL-6*, *TNF-*α, *iNOS*, *IL-10* and *IGF-1* mRNA in quadriceps 2, 5, 10 days after Ctx injury of WT and Mecp2^***-/y***^ mice. All data points were calculated in triplicate, normalized to *GAPDH* and expressed as relative to not injured WT expression. Each time point is compared to the No Ctx situation of the same genotype and significance is calculated with t test. Data are represented as mean ± s.e.m. (*IL-6 P* value: ** = 0,0027, * = 0,0277; *TNF-*α *P* value: ** = 0,0033, * = 0,0195, ****<0,0001; *iNOS P* value: * = 0.0215, * = 0,0443, * = 0,0210; *IL-10 P* value: ** = 0,0089, *** = 0,0009, ****<0,0001; *IGF-1 P* value: * = 0,0477, * = 0,0191; n = 3).

To analyze whether MeCP2 expression is required for regeneration of adult muscle, we acutely injured the tissue by injecting gastrocnemius or quadriceps muscles of 6 weeks old *Mecp2* ^*-/y*^ mice and WT littermates with cardiotoxin (Ctx). Muscles were dissected 2, 5 and 10 days after injury and processed for either histology or mRNA expression analysis. The regeneration process was similar in WT and mutant mice, with transient infiltration of the tissue by inflammatory leukocytes, appearance of regenerating centronucleated myofibers and timely disposal of necrotic debris (Fig 3A, top panels). CSA of regenerating myofibers was significantly lower in injured muscles of *Mecp2* ^*-/y*^ mice compared to WT littermates both 5 and 10 days after damage (Fig 3A, bottom panels; 3B). These results indicate that also during regeneration fiber growth is affected by MeCP2 deficiency.

Macrophages (MPs) are the major infiltrating population of phagocytes in the injured muscle and are an important source of cytokines, chemokines and growth factors. Immunohistochemical analyses of *Mecp2*-null and WT regenerating muscles from 6 weeks old mice indicate that the extent of macrophage infiltration and the number of macrophages expressing markers of conventional or alternative activation (CD86 or CD206, respectively) are similar (Fig 3C). However, the expression profile of cytokines and macrophage specific enzymes induced by injury differs between the two genotypes. In particular, MeCP2 deficiency, while not affecting the level of expression of the inflammatory markers IL6, TNF-α, iNOS and IL10, alters their kinetic of activation, generally delaying it (Fig 3D). Conversely, levels of IGF-1 remain consistently lower in *Mecp2* ^*-/y*^ muscles, confirming that lack of MeCP2 is associated with IGF-1 deficiency (Fig 3D).

### Muscles specifically ablated for *Mecp2* do not show growth defects

**Fig 4:**
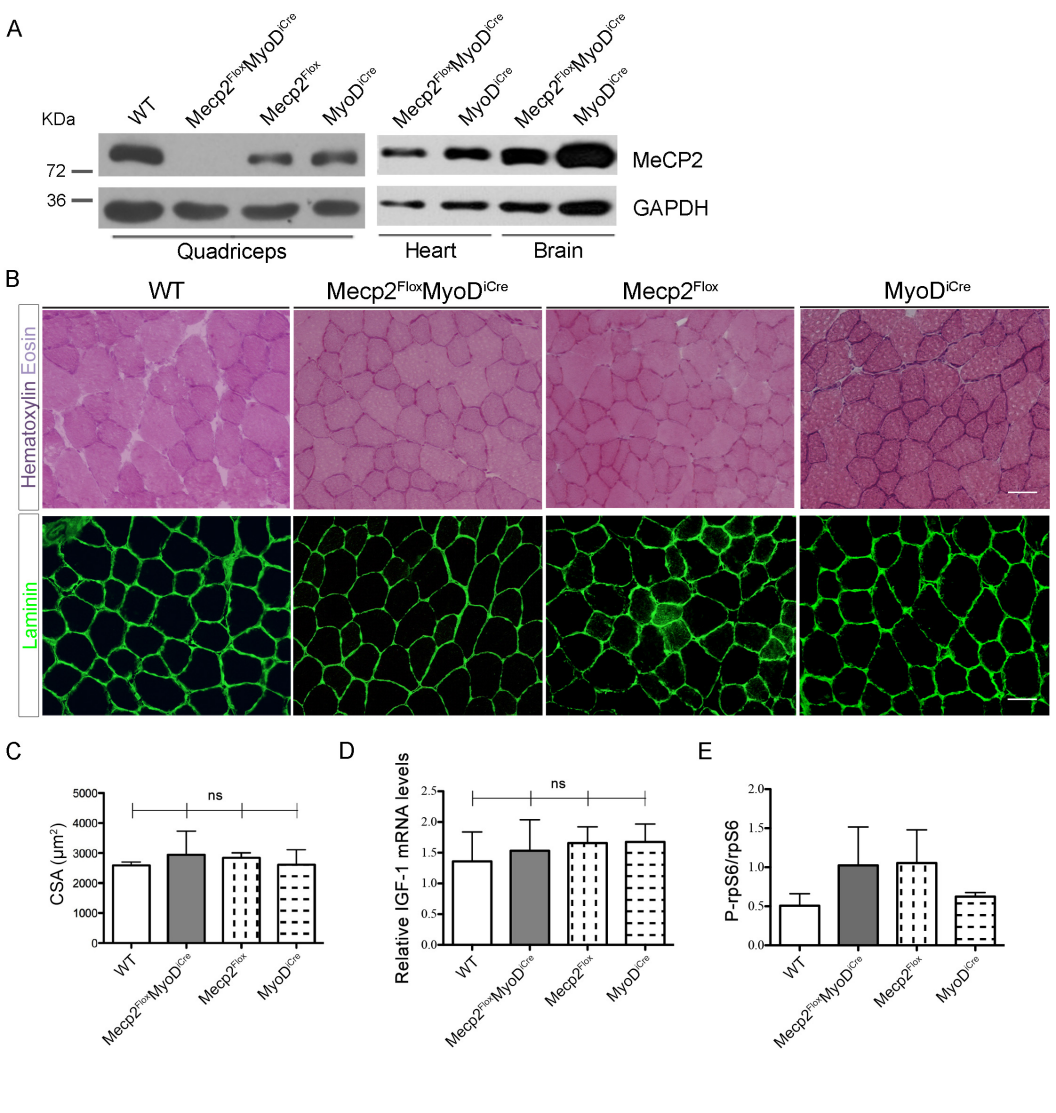
Muscle specific deletion of *Mecp2* does not lead to the observed muscular alterations. (A) WBs confirm the specific skeletal muscle deletion of *Mecp2* in the transgenic *Mecp2* ^***Flox***^ *MyoD* ^***iCre***^ mice. WT, *Mecp2* ^***Flox***^ and *MyoD* ^***iCre***^ genotypes are used as controls. (B) Representative TA cross-sections stained with Hematoxylin and Eosin (upper panels) or immunostained for Laminin (bottom panels). Scale bar = 50 μm. (C) Mean CSA ± s.e.m. showing no statistically significant differences between *Mecp2* ^***Flox***^ *MyoD* ^***iCre***^ mice fibers and control ones. At least 130 myofibers were counted for each animal. Significance was calculated using one-way ANOVA (ns: 0.8520). (D) *Mecp2* ^***Flox***^ *MyoD* ^***iCre***^ mice and controls show similar levels of Gast *IGF-1* mRNA. Significance was calculated using one-way ANOVA (n≥3; ns: 0.9283). (E) Graph showing similar levels of P-rpS6 between *Mecp2* ^***Flox***^ *MyoD* ^***iCre***^ quadriceps and the corresponding controls. Data are represented as mean ± s.e.m. (n = 2).

So far, our results suggest that fiber growth is affected in the absence of MeCP2; however, they do not permit to address whether the observed phenotype is cell- or non cell-autonomous. To get insight into the muscle-specific role of MeCP2, we crossed heterozygous *Mecp2* ^flox/+^ female mice [10,22] with *MyoD* ^*iCre*^ males [23] thus obtaining mice with the deletion of *Mecp2* in all myoblasts and muscle fibers. *Mecp2* ^flox/y^;*MyoD* ^*iCre*^ mice do not spontaneously develop RTT and are fertile. WB analyses confirmed that muscles of adult *Mecp2* ^flox/y^;*MyoD* ^*iCre*^ mice do not express MeCP2, while the expression is conserved in other organs and tissues. In accordance with a previous publication, we observed reduced MeCP2 expression in *Mecp2* ^*flox*^ animals (Fig 4A) [24].

Histological and morphometrical analyses, performed on 5 months old mice, revealed that *Mecp2* ^*flox/y*^ *;MyoD* ^*iCre*^ TA muscles have normal structure and myofibers, and comparable CSA with respect to control littermates (Fig 4B and 4C). IGF-1 expression in *Mecp2* ^*flox/y*^ *;MyoD* ^*iCre*^ muscle is conserved (Fig 4D) and no alteration of the P-rpS6 content is evident (Fig 4E). The regeneration of the adult *Mecp2* ^*flox/y*^ *;MyoD* ^*iCre*^ muscle is similar to that of control mice, and quantification of mean myofiber CSA 5 days after damage does not reveal statistically significant differences (S3 Fig).

Overall, these results indicate that skeletal muscles do not directly require MeCP2 for development and growth. Accordingly, *Mecp2* ^*flox/y*^ *;MyoD* ^*iCre*^ muscles are not hypotrophic, as indicated by the similar skeletal muscle weight of *Mecp2* ^flox/y^;MyoD^iCre^ mice and controls at various postnatal ages (S1 Table).

## Discussion

So far, RTT research has focused mainly on the nervous system and genetic deletion studies have shown that inactivation of *Mecp2* in post-mitotic neurons leads to several symptoms common with those of *Mecp2-*null mice. However, the concept of RTT as an exclusively neuronal disease is changing, therefore, highlighting the relevance of searching for tissue dysfunctions associated with the absence of MeCP2.

RTT patients are characterized by a significant hypotonia; concordantly, the *Mecp2*-null mouse model is characterized by a decreased growth and severe hypotonia. Skeletal muscles account for approximately 40% of the total body mass and represent a major player in energy balance. Muscle fiber size changes physiologically in function of the environmental demand and it adapts to the various pathological conditions. One of the main mechanisms of this dynamics is the regulation of protein homeostasis obtained through the balance between protein synthesis and degradation [25]. Here we show that MeCP2 deficiency leads to a severe muscle hypotrophy by regulating myofiber size in a non cell-autonomous way. Indeed, while the *Mecp2*-null mice are characterized by a reduced skeletal muscle mass, the novel *Mecp2* ^*flox/y*^ *;MyoD* ^*iCre*^ transgenic line, in which *Mecp2* has been deleted only in myoblasts and skeletal muscle fibers, does not show any of the RTT-like myopathy: in particular, weight and size of skeletal muscles do not differ from those of control littermates. Of relevance, this is the first genetic mouse model in which *Mecp2* has been ablated in a tissue different from the nervous system, and our study suggests that *Mecp2* is not directly required for proper development, growth and regeneration of skeletal muscle.

The muscle phenotype observed in the null mice could be caused either by neuromuscular plaque defects and/or by an altered crosstalk of paracrine/endocrine signaling. Previous electromyography studies on RTT patients have shown a normal corticospinal pathway [11], therefore dismissing the neuromuscular plaque as a major player in the observed MeCP2-linked muscular phenotype. Accordingly, our analyses of the number and characteristics of the neuromuscular junctions did not reveal any overt difference in *Mecp2-*null mice with respect to WT littermates. Thus, altered growth signaling might determine the muscle defects characterizing the *Mecp2*-null mice.

The Growth Hormone (GH)/IGF1 axis is a critical regulator of somatic growth and other developmental processes [26] *via* the production and secretion of hepatic IGF-1, which then exerts its biological effects on the other tissues. One of the major target tissues is the skeletal muscle, where IGF-1 is also produced and is an important regulator of mass both in basal conditions and in response to exercise and muscle injury. IGF-1 administration to *Mecp2-*null mice ameliorates several RTT-features [12]. More importantly, it ameliorates certain breathing and behavioral abnormalities in RTT girls [27]. Here, we have shown that *Mecp2*-null mice have decreased IGF-1 protein levels both in plasma and skeletal muscle. The corresponding mRNA levels are reduced in skeletal muscle, suggesting a local defective expression. In accordance with decreased IGF-1 levels, we found that phosphorylation of rpS6 is significantly reduced in the muscles of *Mecp2*-null mice. Since the levels of IGF-1 and rpS6 phosphorylation are normal in the *Mecp2* ^*flox/y*^ *;MyoD* ^*iCre*^ transgenic line, we suggest that this pathway regulated by paracrine/endocrine factors is one of the major causes of the observed muscle hypotrophy.

During the past years, several genetic mouse models have been used to dissect the signaling pathways involved in muscle-mass regulation and to identify strategies to affect the activity of IGF1-Akt signaling [28]. Although perfectly aware of the vast number of interactions of the IGF1-Akt pathway with others factors and molecular trails, we would like to highlight that in adult skeletal muscle, hypertrophy can be induced by blocking myostatin through post-developmental myostatin gene knockout, with follistatin (a myostatin antagonist) or anti-myostatin antibodies. Fiber size is also controlled by AMP-activated kinases and mTOR, while corticosteroids reduce the production of IGF1 [28]. Mouse models genetically modified in components of these pathways (listed in ref. 28) might be used in future studies to understand the basis of reduced muscle growth in *Mecp2-*null mice.

In addition to IGF-1, skeletal muscle is the source of several myokines secreted by contracting skeletal myocytes that can have an autocrine, paracrine or endocrine activity. Amongst these, there is BDNF, whose circulating levels are determined by both skeletal muscle and brain [29]. BDNF has been reported as a direct target of MeCP2 repression [30,31]; however, its levels are reduced in the brain of RTT patients and *Mecp2* mutant mice and treatment with BDNF have been suggested as a therapeutic approach for RTT [32,33]. Here, we show that the expression of muscle derived *Bdnf* gene is also greatly reduced in *Mecp2*-null mice.

Skeletal muscle homeostasis also depends on the ability of the tissue to initiate an extensive repair process counteracting the loss of muscle mass and fibers, through the activation of myogenic stem cells, the satellite cells, in a regenerative process that recapitulate myogenesis [34]. Following acute muscle damage, *Mecp2*-null mice are able to regenerate new myofibers with proper timing. However, and in accordance with the important reported hypotrophy, new fibers maintain a smaller size with respect to the control littermates. Since muscle regeneration and myofiber size are normal in mice with muscle specific *Mecp2* inactivation, *Mecp2* appears to be dispensable for myogenesis in satellite cells while the observed fiber growth defects in the null animals depend on paracrine/endocrine signals. Indeed, IGF-1 levels remain low throughout the entire regeneration process.

A well-timed inflammatory response and corresponding cytokine production is also crucial for proper generation of new muscle fibers. Inflammatory macrophages infiltrate the acutely injured tissue, secrete inflammatory signals, such as TNF-α, clear myofibers and undergo a switch towards a reparatory program. At later times, alternatively activated macrophages accumulate into injured muscles and actively sustain fiber reconstitution mainly by generating signals with trophic functions, such as IGF-1 or IL-10. In the muscles of *Mecp2*-null mice, infiltration of macrophages appears to occur properly, and the balance between inflammatory and alternatively activated macrophages is similar with respect to control mice. Nevertheless, when we compared the kinetics of expression of several cytokines, we found that *Mecp2*-null muscles show a delayed appearance of both inflammatory and anti-inflammatory signals after damage, with the notable exception of IGF-1, whose expression levels are always lower in the MeCP2 deficient mice. These results suggest that altered kinetics of activation of cytokines and macrophage markers may contribute, together with the global IGF-1 deficiency, to the regenerating fiber growth defect. Of note, while we were revising this manuscript, it has been demonstrated that *Mecp2* is expressed and required by macrophages for a proper response to inflammatory stimuli [35]. The authors have suggested that the *Mecp2*-null macrophage altered responsiveness to environmental stimuli might affect tissue homeostasis, therefore contributing to RTT clinical features occurring in multiple organ systems. Since macrophages have been proposed as one of the major source of IGF-1 [36] and the functional interference with IGF-1 is known to influence the characteristics of the inflammatory response in skeletal muscle [37,38], we raise the possibility that the observed altered response to muscular injury and the IGF-1 deficiency might be causally related. Further studies are necessary to address this contention.

In conclusion we have demonstrated that a general deficiency of MeCP2 causes an important muscle hypotrophy that is probably the main cause of hypotonia characterizing most RTT patients. This defect appears to be mostly due to non cell-autonomous mechanisms related to paracrine/endocrine signaling and to the synthesis of trophic factors such as IGF-1 and BDNF. The fact that the down-regulation of these trophic factors, as reported in previous literature and herein, does not depend directly on MeCP2 deficiency, underscores the relevance of identifying the upstream molecular pathway(s) causing this common alteration. Restoring this pathway(s) might, in fact, represent a more effective therapeutic strategy with respect to directly altering BDNF or IGF-1 levels.

## Materials and Methods

### Animals

*Mecp2*-null males (*Mecp2* ^*-/y*^) B6.129P2(C)Mecp2^tm1.1 Bird^/J [10] together with the WT C57BL/6J controls were obtained by crossing heterozygous females (*Mecp2* ^*-/+*^) to WT C57BL/6J males. This mouse line was housed at Charles River (Calco, Lecco, Italy). The muscle-specific *Mecp2*-conditional mouse line was obtained by crossing C57BL/6J *Mecp2* ^flox/+^ [10] with FVB-Tg *MyoD* ^*iCre*^ mice [23]. Breeding of this mouse line was done in the animal facility at San Raffaele Scientific Institute. All experimental protocols were approved by the San Raffaele Scientific Institutional Animal Care and Use Committee (IACUC 355, 489, 450 and 610) in accordance with the Italian law.

Animals were anesthetized with 2-Bromo-2-chloro-1,1,1-trifluoroethane or Avertin and sacrificed by cervical dislocation. All efforts were made to minimize suffering.

### Organs and body weight analysis

Vital organs such as heart, kidneys, liver, lung and spleen were recovered. All of them were observed macroscopically and their appearance was compared between the different genotypes. Body and organs weights were measured and recorded.

### Acute muscle damage

Animals were anaesthetized by inhalation with 2-bromo-2-chloro-1,1,1-trifluoroethane, ≥99% (B4388-125ML, Sigma-Aldrich, Saint Louis, MO, USA) and subsequently injected with Ctx (obtained from *Naja mossambica mossambica* snake, C9759 Sigma-Aldrich): 50 μl Ctx 10μM for WT and *Mecp2* ^*-/y*^ quadriceps and gastrocnemii; 50 μl Ctx 15μM for *Mecp2* ^flox/y^ *MyoD* ^*iCre*^ and controls tibialis anterior (TA). Mice were sacrificed and muscles harvested 2, 5 or 10 days after injection.

### qPCR

Total RNA was isolated from gastrocnemii or quadriceps using the RNeasy Fibrous Tissue Mini Kit (74704, QIAGEN, GmbH, Germany) as instructed by the manufacturer. Extracted RNA was quantified with NanoDrop (ND-1000 Spectrophotometer EuroClone, Milan, Italy) and its quality was assessed through a denaturing 1% agarose gel with EtBr. Complementary DNA (cDNA) was transcribed using the RT^2^ First Strand Kit (330401, QIAGEN) according to the manufacturer’s instructions. PCR reactions were run on a StepOnePlus Real-Time PCR System (Applied Biosystems, Waltham, MA, USA), using SYBR Select Master Mix (4472908, Applied Biosystems) and primers (200 nM). ΔCt were calculated using *GAPDH* as normalizer. Gene expression was represented as relative to the WT one.

Primers list: *GAPDH*: Fwd: 5’-CACCATCTTCCAGGAGCGAG-3’, Rev: 5’-CCTTCTCCATGGTGGTGAAGAC-3’; *Collagen I*: Fwd: 5’-GCTCCTCTTAGGGGCCACT-3’, Rev: 5’-CCACGTCTCACCATTGGGG-3’; *IGF-1*: Fwd: 5’-CTCTGCTTGCTCACCTTCAC-3’, Rev: 5’-CTCATCCACAATGCCTGTCT-3’; *IGF-1R*:

Fwd: 5’-ACACCAGGAACAACGGAGAG-3’, Rev 5’-AGGCAGGTCTACATCCACCA-3’; *BDNF*: Fwd: 5’-AAGTCTGCATTACATTCCTCGA-3’; Rev: 5’-TTATCAATTCACAATTAAAGCAGCAT-3’; *IL-6*: Fwd: 5’-CTCTGCAAGAGACTTCCATCCAGT-3’, Rev: 5’-CGTGGTTGTCACCAGCATCA-3’; *TNF-α*: Fwd: 5’-TCCCAGGTTCTCTTCAAGGGA-3’, Rev: 5’-GGTGAGGAGCACGTAGTCGG-3’; *iNOS*: Fwd: 5’-AGCCAAGCCCTCACCTACTT-3’, Rev: 5’-TCTCTGCCTATCCGTCTCGT-3’; *IL-10*: Fwd: 5’-ATTTGAATTCCCTGGGTGAGAAG-3’, Rev: 5’-CACAGGGGAGAAATCGATGACA-3’

### Western Blotting

Muscles and organs from *Mecp2* ^*-/y*^, *Mecp2* ^flox/y^ *MyoD* ^*iCre*^ and controls were homogenized with Ultra-Turrax (by IKA) in lysis buffer (EDTA 5mM, NaCl 350 mM, Triton X-100 1%, Glycerol 10%, NaF 50 mM, Tris-HCl pH 7.5 20 mM) with PhosSTOP 1X (04906837001, Roche, Basel, Switzerland) and cOmplete EDTA-free protease inhibitor cocktail (11873580001, Roche). Soluble proteins were quantified with Pierce BCA Protein Assay Kit (23227, Thermo Scientific, Waltham, MA, USA). Protein were electrophoresed on 10% SDS-polyacrylamide gel and blotted onto nitrocellulose using the Trans-Blot Turbo Transfer System from Bio-Rad (11 min, 2.5 A, 25 V) and probed with the following primary antibodies: anti-S6 ribosomal protein 5G10 1:1000 (2217, Cell Signaling, Boston, MA, USA), anti-phospho S6 ribosomal protein (Ser240/244) 1:1000 (2215, Cell Signaling), anti-MeCP2 D4F3 1:1000 (3456, Cell Signaling), anti-GAPDH 1:1000 (TAB1001 Thermo Scientific). For Fig 2C, membranes were incubated with the anti-rabbit secondary antibody AlexaFluor 488 (1:2000, A21206 Life Technologies, Boston, MA, USA), then fluorescent bands were detected and visualized using the laser scanner Typhoon FLA 9000 (GE Healthcare, Buckinghamshire, UK). For Fig 4A, HRP-conjugated anti-rabbit secondary antibodies were used (31460, 1:10000, Thermo Scientific) and blots were visualized using a chemiluminescence-based detection system (SuperSignal West Pico Chemiluminescent Substrate, Thermo Scientific). For quantitative measurement, blots were analyzed with ImageJ, normalizing band intensities to GAPDH levels. To evaluate rpS6 phosphorylation we made a ratio between the normalized phospho-protein and the normalized total isoforms of the same protein.

### ELISA Assay

To determinate Insulin-like Growth Factor I (IGF-1) concentrations in plasma and triceps homogenates of 6 weeks old WT and *Mecp2* ^*-/y*^ mice, we employed the Quantikine ELISA Mouse/Rat IGF-1 Immunoassay (MG100, R&D Systems, Minneapolis, MN, USA), according to the manufacturer’s instructions. Optical density was determined using the VersaMax Tunable Microplate Reader.

### Histology and Immunofluorescence

Muscles were collected and embedded in OCT or directly frozen in liquid nitrogen cool isopentane. 8 micron serial muscle sections were stained with Hematoxylin and Eosin (H&E) (Sigma-Aldrich) and Sirius Red (Sigma-Aldrich) according to standard procedures. For quantification of Sirius Red areas the images were subsequently analyzed using the batch mode of the ImageJ vs 1.48 macro. This macro was written to quantify the percentage of fibrosis compared to the total amount of tissue within an image. The color threshold algorithm used by this macro is based on an algorithm written by G. Landini (version v1.8) available at [www.mecourse.com/landinig/software/software.htm].

Immunofluorescence on frozen section muscles were generally carried out as in [39]. For macrophages and fiber type specific antibodies, muscle sections were fixed with 4% paraformaldehyde in PBS. After 10 minutes in glycine 0.1M, they were blocked for 1 hour in 5% serum (FBS for CD68 staining, donkey serum for CD86 and CD206 staining, FCS for MHCI and MHCIIB staining), 0.1% Triton X-100, 5% BSA solution in PBS before incubation with primary antibodies in a 5% BSA in PBS solution. Incubation was performed O.N. at 4°C.

Primary antibodies: chicken anti-laminin (1:500, Sigma-Aldrich), rabbit anti-synaptophysin (1:500, Invitrogen, Boston, MA, USA), mouse anti-neurofilament (1:150, Sigma-Aldrich); tetramethylrhodamine α-bungarotoxin (1:100 Invitrogen); rat anti-CD68 (1:150; AbD Serotec, Bio Rad, Hercules, CA, USA), rat anti-CD206 (1:100; AbD Serotec), rat anti-CD86 (1:100; BD-Pharmingen, Franklin Lakes, NJ, USA), mouse IgG2b anti-MHCI BAD5 (1:50), mouse IgM anti-MHCIIB BF-F3 (1:50) [40,41]. Appropriate Alexa Fluor (Alexa 488 or Alexa 546)-conjugated antibodies (1:500; Invitrogen) were used as second-step reagent. Nuclei were counterstained with DAPI (1:1000, Sigma Aldrich) and mounted with Fluorescence Mounting Medium (Dako, Glostrup, Denmark).

### Measurement of myofiber cross sectional area (CSA)

Cross sectional area of the myofibers was calculated on Laminin stained section images obtained from TA and Gast muscles of mice. Measurement of the area was performed using the analyzer software ImageJ vs. 1.48.

### Image acquisition and manipulation

Fluorescent and phase contrast images were taken using Nikon Eclipse E600 microscope. Image acquisition was done using the Nikon digital camera DXM1200 and the acquisition software Nikon ACT-1 (lenses Plan Fluor:X4/0.13, X10/0.33, X20/0.50, X40/0.75).

### Statistics

Statistical significance of variations among the different experimental groups was determined by two-tailed Student's t test (Prism 5, GraphPad Software) or One-way ANOVA (Prism 5, GraphPad Software). P values less than 0.05 were considered significant.

## Supporting Information

S1 Fig Muscle fiber type composition is similar between WT and *Mecp2*-null mice. (A) Representative WT and *Mecp2*-null gastrocnemius muscle sections immunostained with BAD5 and BF-F3 to recognize, respectively, fibers I (MHCI) and IIb (MHCIIB). Scale bar = 50 μm. (B) Quantification of the number of type I and type IIb fibers. At least 200 myofibers were counted for each animal, n = 3 per genotype. Data are represented as mean ± s.e.m. Significance is calculated with *t* test (ns, P value: 0.9763).
(TIF)

S2 Fig Density of neuromuscular junctions is similar between WT and *Mecp2-*null mice. Representative WT and *Mecp2*-null tibialis anterior muscle sections stained with α-bungarotoxin, Ab against synaptophysin and neurofilament, DAPI. Scale bars = 50 μm
(TIF)

S3 Fig Regeneration after acute muscle damage in *Mecp2* ^*Flox*^ *MyoD* ^*iCre*^ skeletal muscle. (A) Representative TA cross-sections of *Mecp2* ^*Flox*^ *MyoD* ^*iCre*^ and control mice 5 days after Ctx injury, stained with Hematoxylin and Eosin or immunostained for Laminin and DAPI to recognize centrally-nucleated myofibers. Scale bar = 50 μm. (B) Mean CSA ± s.e.m. of regenerating centronucleated myofibers shows no statistically significant differences between the different genotypes. At least 150 regenerating centrally-nucleated myofibers were counted for each animal. Significance was calculated using one-way ANOVA (ns: 0.1116).
(TIF)

S1 Table Weights of muscles and organs retrieved from *Mecp2* ^*Flox*^ *MyoD* ^*iCre*^ and control mice at months 5 and 3, and 6 weeks of age. Each genotype was compared with the WT and significance was calculated with t test (* p< 0,05, ** p<0,01). Data are indicated as means ± s.e.m. n = 4 per genotype at each time point.
(DOCX)
