# Supplementary figures and images for: MeCP2 Affects Skeletal Muscle Growth and Morphology through Non Cell-Autonomous Mechanisms
Source: PLoS One. 2015 Jun 22;10(6):e0130183. doi: 10.1371/journal.pone.0130183 (PMC4476581; doi:10.1371/journal.pone.0130183)

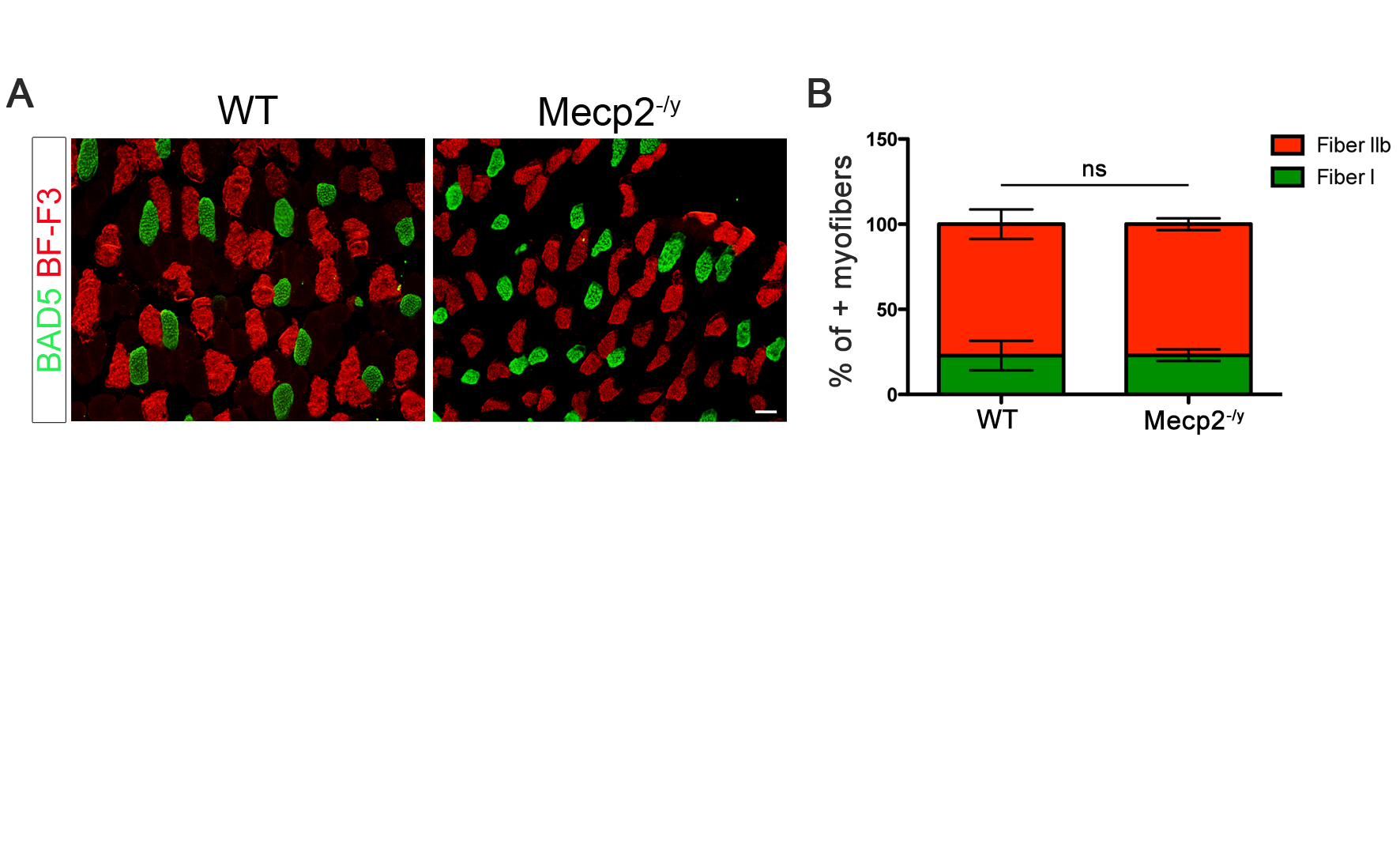

Supplement: S1 Fig — (A) Representative WT and Mecp2-null gastrocnemius muscle sections immunostained with BAD5 and BF-F3 to recognize, respectively, fibers I (MHCI) and IIb (MHCIIB). Scale bar = 50 μm. (B) Quantification of the number of type I and type IIb fibers. At least 200 myofibers were counted for each animal, n = 3 per genotype. Data are represented as mean ± s.e.m. Significance is calculated with t test (ns, P value: 0.9763). (TIF) [file pone.0130183.s001.tif]

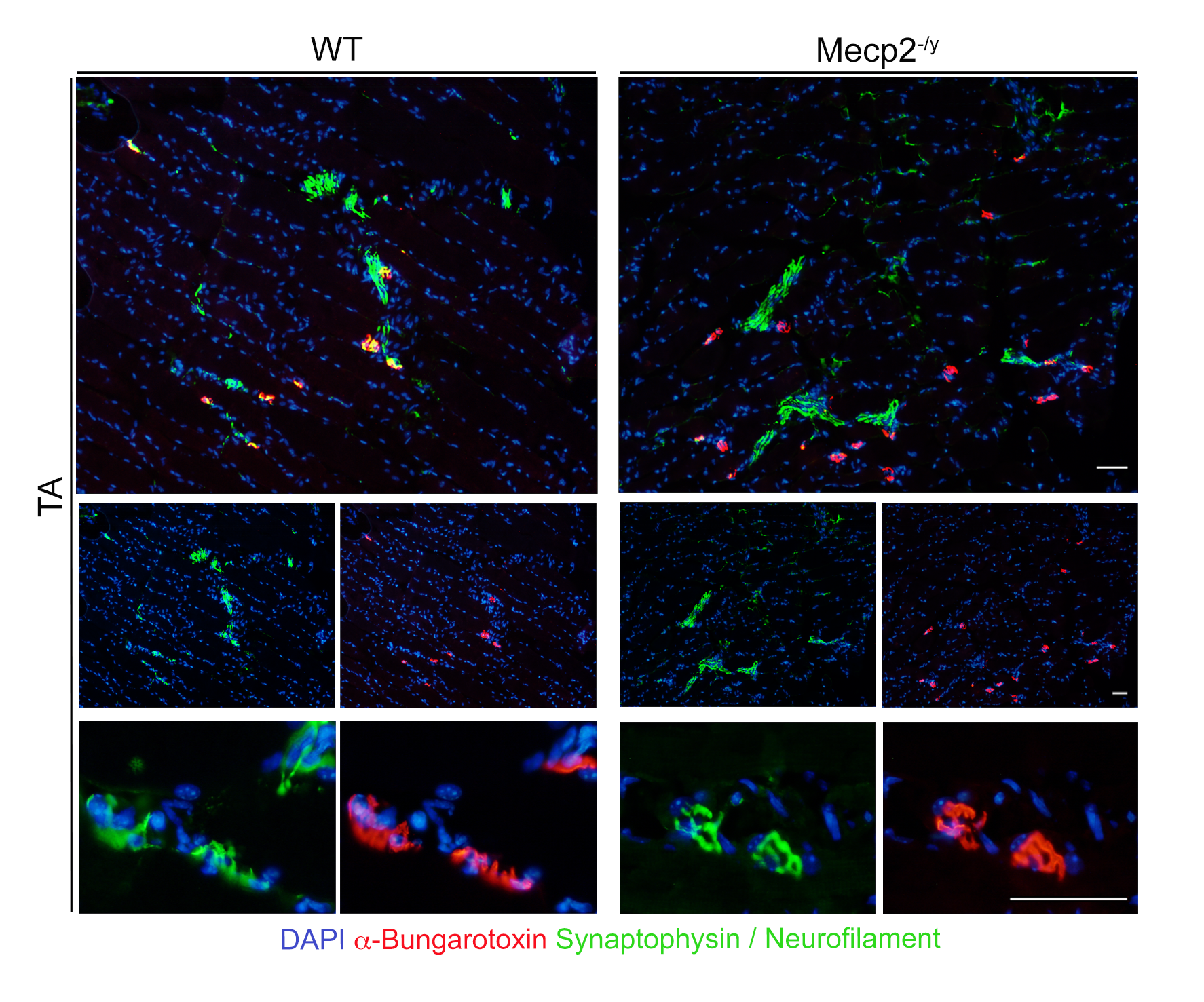

Supplement: S2 Fig — Representative WT and Mecp2-null tibialis anterior muscle sections stained with α-bungarotoxin, Ab against synaptophysin and neurofilament, DAPI. Scale bars = 50 μm (TIF) [file pone.0130183.s002.tif]

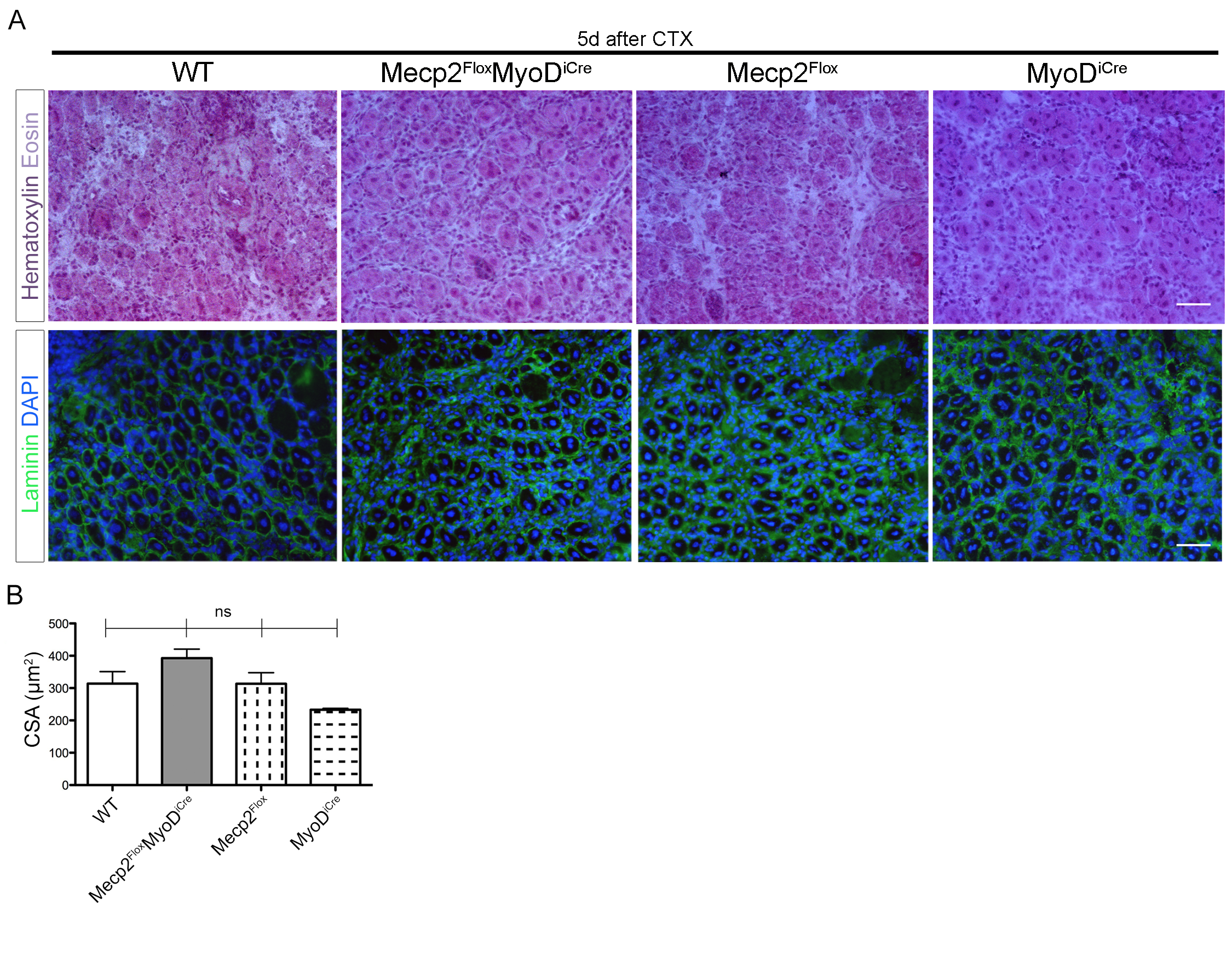

Supplement: S3 Fig — (A) Representative TA cross-sections of Mecp2 Flox MyoD iCre and control mice 5 days after Ctx injury, stained with Hematoxylin and Eosin or immunostained for Laminin and DAPI to recognize centrally-nucleated myofibers. Scale bar = 50 μm. (B) Mean CSA ± s.e.m. of regenerating centronucleated myofibers shows no statistically significant differences between the different genotypes. At least 150 regenerating centrally-nucleated myofibers were counted for each animal. Significance was calculated using one-way ANOVA (ns: 0.1116). (TIF) [file pone.0130183.s003.tif]
