# Supplementary material for: MeCP2 Affects Skeletal Muscle Growth and Morphology through Non Cell-Autonomous Mechanisms
Source: PLoS One. 2015 Jun 22;10(6):e0130183. doi: 10.1371/journal.pone.0130183 (PMC4476581; doi:10.1371/journal.pone.0130183)
Supplement: S1 Table — Each genotype was compared with the WT and significance was calculated with t test (* p< 0,05, ** p<0,01). Data are indicated as means ± s.e.m. n = 4 per genotype at each time point. (DOCX) [file pone.0130183.s004.docx]

| **5 months** | **WT (gr)** | **Mecp2^Flox^MyoD^iCre^ (gr)** | **Significance** | **Mecp2^Flox^ (gr)** | **Significance** | **MyoD^iCre^ (gr)** | **Significance** |
| --- | --- | --- | --- | --- | --- | --- | --- |
| Tibialis anterior | 0,083 ± 0,007 | 0,067 ± 0,008 | ns | 0,084 ± 0,005 | ns | 0,078 ± 0,007 | ns |
| Gastrocnemius | 0,240 ± 0,012 | 0,215 ± 0,009 | ns | 0,205 ± 0,006 | * | 0,225 ± 0,013 | ns |
| Quadriceps | 0,213 ± 0,032 | 0,202 ± 0,021 | ns | 0,239 ± 0,011 | ns | 0,221 ± 0,013 | ns |
| Triceps | 0,146 ± 0,011 | 0,127 ± 0,009 | ns | 0,138 ± 0,006 | ns | 0,146 ± 0,006 | ns |
| Heart | 0,199 ± 0,014 | 0,189 ± 0,011 | ns | 0,195 ± 0,009 | ns | 0,208 ± 0,008 | ns |
| Spleen | 0,135 ± 0,014 | 0,106 ± 0,006 | ns | 0,109 ± 0,010 | ns | 0,109 ± 0,006 | ns |
| Kidney | 0,285 ± 0,016 | 0,246 ± 0,017 | ns | 0,248 ± 0,013 | ns | 0,259 ± 0,021 | ns |
| Liver | 1,918 ± 0,169 | 1,593 ± 0,142 | ns | 1,572 ± 0,109 | ns | 1,647 ± 0,157 | ns |
| Brain | 0,509 ± 0,011 | 0,439 ± 0,015 | ** | 0,450 ± 0,014 | * | 0,496 ± 0,013 | ns |
| Total Weight | 36,81 ± 2,69 | 30,72 ± 2,22 | ns | 29,05 ± 0,47 | * | 32,62 ± 2,34 | ns |
| **3 months** | **WT (gr)** | **Mecp2^Flox^MyoD^iCre^ (gr)** | **Significance** | **Mecp2^Flox^ (gr)** | **Significance** | **MyoD^iCre^ (gr)** | **Significance** |
| Tibialis anterior | 0,069 ± 0,011 | 0,076 ± 0,015 | ns | 0,070 ± 0,012 | ns | 0,075 ± 0,010 | ns |
| Gastrocnemius | 0,212 ±0,024 | 0,187 ± 0,035 | ns | 0,192 ± 0,031 | ns | 0,206 ± 0,030 | ns |
| Quadriceps | 0,223 ± 0,031 | 0,217 ± 0,061 | ns | 0,191 ± 0,052 | ns | 0,182 ± 0,081 | ns |
| Triceps | 0,128 ± 0,025 | 0,204 ± 0,075 | ns | 0,117 ± 0,031 | ns | 0,143 ± 0,012 | ns |
| Heart | 0,177 ±0,034 | 0,191 ± 0,023 | ns | 0,154 ± 0,028 | ns | 0,175 ± 0,032 | ns |
| Spleen | 0,121 ± 0,031 | 0,106 ± 0,021 | ns | 0,110 ± 0,036 | ns | 0,118 ± 0,012 | ns |
| Kidney | 0,231 ± 0,040 | 0,215 ± 0,030 | ns | 0,200 ± 0,029 | ns | 0,242 ± 0,024 | ns |
| Liver | 1,865 ± 0,502 | 1,587 ± 0,286 | ns | 1,568 ± 0,165 | ns | 1,593 ± 0,201 | ns |
| Brain | 0,464 ± 0,039 | 0,452 ± 0,018 | ns | 0,426 ± 0,061 | ns | 0,473 ± 0,030 | ns |
| Total Weight | 30,88 ± 2,64 | 26,42 ± 2,28 | * | 26,46 ± 5,14 | ns | 28,47 ± 1,46 | ns |
| **6 weeks** | **WT (gr)** | **Mecp2^Flox^MyoD^iCre^ (gr)** | **Significance** | **Mecp2^Flox^ (gr)** | **Significance** | **MyoD^iCre^ (gr)** | **Significance** |
| Tibialis anterior | 0,057 ± 0,008 | 0,064 ± 0,015 | ns | 0,059 ± 0,004 | ns | 0,072 ± 0,022 | ns |
| Gastrocnemius | 0,138 ± 0,024 | 0,168 ± 0,034 | ns | 0,148 ± 0,019 | ns | 0,193 ± 0,041 | ns |
| Quadriceps | 0,146 ± 0,029 | 0,135 ± 0,051 | ns | 0,124 ± 0,040 | ns | 0,171 ± 0,058 | ns |
| Triceps | 0,083 ± 0,029 | 0,092 ± 0,016 | ns | 0,180 ± 0,156 | ns | 0,104 ± 0,016 | ns |
| Heart | 0,114 ± 0,011 | 0,145 ± 0,024 | ns | 0,130 ± 0,014 | ns | 0,138 ± 0,012 | ns |
| Spleen | 0,100 ±0,018 | 0,100 ± 0,014 | ns | 0,089 ± 0,010 | ns | 0,095 ± 0,013 | ns |
| Kidney | 0,176 ± 0,035 | 0,234 ± 0,046 | ns | 0,182 ± 0,012 | ns | 0,226 ± 0,045 | ns |
| Liver | 1,190 ± 0,186 | 1,575 ± 0,189 | * | 1,293 ± 0,245 | ns | 1,583 ± 0,169 | * |
| Brain | 0,439 ± 0,062 | 0,475 ± 0,068 | ns | 0,438 ± 0,016 | ns | 0,490 ± 0,018 | ns |
| Total Weight | 20,88 ± 3,79 | 23,98 ± 1,49 | ns | 22,23 ± 1,61 | ns | 24,56 ± 1,67 | ns |
